# Harnessing the power of biosensors for environmental monitoring of pesticides in water

**DOI:** 10.1007/s00253-025-13461-x

**Published:** 2025-04-12

**Authors:** Filipa Mendes, Beatriz O. Machado, Bruno B. Castro, Maria João Sousa, Susana R. Chaves

**Affiliations:** 1 Centre of Molecular and Environmental Biology (CBMA) & Aquatic Research Network (ARNET), Department of Biology, School of Sciences of the University of Minho, 4710-057 Braga, Portugal; 2 Institute of Science and Innovation for Bio-Sustainability (IB-S), School of Sciences of the University of Minho, 4710-057 Braga, Portugal

**Keywords:** Pesticides, Aquatic ecosystems, Biosensors, Environmental monitoring, Water quality

## Abstract

**Abstract:**

The current strong reliance on synthetic chemicals, namely pesticides, is far from environmentally sustainable. These xenobiotics contribute significantly to global change and to the current biodiversity crisis, but have been overlooked when compared to other agents (e.g., climate change). Aquatic ecosystems are particularly vulnerable to pesticides, making monitoring programs essential to preserve ecosystem health, safeguard biodiversity, ensure water quality, and mitigate potential human health risks associated with contaminated water sources. Biosensors show great potential as time/cost-effective and disposable systems for the high-throughput detection (and quantification) of these pollutants. In this mini-review, we provide an overview of biosensors specifically developed for environmental water monitoring, covering different pesticide classes (and active ingredients), and types of biosensors (according to the bio-recognition element) and transducers, as well as the nature of sample matrices analyzed. We highlight the variety of biosensors that have been developed and successfully applied to detection of pesticides in aqueous samples, including enzymatic biosensors, immunosensors, aptasensors, and whole cell–based biosensors. While most biosensors have been designed to detect insecticides, expanding their compound target range could significantly streamline monitoring of environmental contaminants. Despite limitations related to stability, reproducibility, and interference from environmental factors, biosensors represent a promising and sustainable technology for pesticide monitoring in the aquatic environments, offering sensitivity and specificity, as well as portability and real-time results. We propose that biosensors would be most effective as an initial screening step in a tiered assessment, complementing conventional methods.

**Key points:**

• *Pesticides harm aquatic ecosystems and biodiversity, requiring better monitoring*

• *Biosensors offer cost-effective solutions to detect pesticides in water samples*

• *Biosensors complement conventional methods as a sustainable tool for initial screens*

## Introduction

Pesticides are substances or biological agents (e.g., *Bacillus thuringiensis* (Angus 1953)), intentionally released into the environment to prevent, control, or destroy pests and diseases. They can be classified according to origin (natural or synthetic) and composition (inorganic or organic), but are most frequently functionally categorized according to their target organism into insecticides, herbicides, fungicides, bactericides, rodenticides, nematicides, and algicides (Mahmood et al. 2016). Pesticides are employed in agriculture to increase crop yields and, consequently, food production. They are also used in public health protection programs to prevent illnesses transmitted by vectors, such as malaria, dengue fever, and schistosomiasis. Additionally, pesticides are applied to improve and maintain non-agricultural areas, including public urban green spaces and sports fields (Sarwar 2015; Nishant and Upadhyay 2016). However, the current strong reliance on pesticides is far from environmentally sustainable, and thus public concerns about their detrimental effects on ecosystems and human health have increased.

Pesticides enter the environment when applied to a target area or when they are disposed of. Once released, the environmental fate of these chemicals can be affected by many factors, such as the properties of the soil (hydraulic loading, organic matter content, particle size distribution) and the intrinsic properties of the pesticide, namely its half-life, water solubility, adsorption coefficient, and volatility (Gavrilescu 2005; Kerle et al. 1994). Pesticides can also move from target sites to other areas through leaching, adsorption, spray drift, volatilization, and runoff, thereby migrating across soil, air, and groundwater (de Araújo et al. 2020; Dad et al. 2022). Indeed, it is estimated that only 0.1% of applied pesticides reach their target site, while the main portion is lost via spray drift, run-off, and accumulation in off-target sites (Parween et al. 2016). Apart from highly inefficient, pesticide application ultimately leads to accumulation of pesticide residues in the atmosphere, soil, water, and biota. Aquatic ecosystems are the main sink for these residues, and aquatic life is particularly vulnerable to their effects (Stehle and Schulz 2015a; de Souza et al. 2020; Rumschlag et al. 2020). The vulnerability of aquatic ecosystems to pesticide pollution is explicitly recognized in the Directive 2009/128/EC of the European Parliament and of the Council of 21 October 2009, which establishes a framework for Community action to achieve the sustainable use of pesticides.

Overall, it has been demonstrated that the negative impact of pesticides on non-target aquatic organisms ultimately affects biodiversity (Malaj et al. 2014; Brühl and Zaller 2019). Along with other synthetic chemicals, pesticides are important agents of global change (Bernhardt et al. 2017), but may have been overlooked as drivers of biodiversity loss when compared to others (Bernhardt et al. 2017; Sigmund et al. 2023). Therefore, the current risk assessment framework for pesticides in aquatic environments would greatly benefit from expanded monitoring programs, an invaluable tool to preserve ecosystem health, safeguard biodiversity, ensure water quality, and mitigate potential human health risks associated with contaminated water sources. Continuous and high-throughput monitoring enables timely interventions when levels surpass acceptable limits, while long-term monitoring provides crucial data to identify trends in pesticide contamination, recognize emerging concerns, and evaluate the efficacy of management practices and regulatory measures.

Given the widespread presence of pesticides in surface (Stehle and Schulz 2015b; Lundqvist et al. 2019; Kruć-Fijałkowska et al. 2022; Laicher et al. 2022; Monticelli Barizon et al. 2022; Chow et al. 2023) and ground (Gonçalves et al. 2007; Chaza et al. 2018; Bexfield et al. 2021) water, monitoring programs would greatly benefit from time- and cost-effective solutions that could complement conventional analytical methods. Biosensors show great potential as disposable systems for the high-throughput detection (and quantification) of these pollutants, especially for screening of a large number of samples. Here, we provide an overview of biosensors that have been specifically developed for environmental water monitoring, covering different pesticide classes and biosensor types. We aim to demonstrate that biosensors represent a promising and sustainable technology for pesticide monitoring in the aquatic environment, offering sensitivity and specificity, as well as portability and real-time results.

## Monitoring pesticides in water

A study that compiled data on worldwide pesticide occurrence in surface waters reported individual pesticide concentrations between 7 ng L^−1^ and 121 µg L^−1^ in rivers and lakes (Mojiri et al. 2020). In wastewaters, concentrations ranged from 23 ng L^−1^ to 3.2 µg L^−1^, similar to levels observed in groundwater (20 ng L^−1^ to 1.1 µg L^−1^), but lower than those in drinking water (141 ng L^−1^ to 14.6 µg L^−1^) (Mojiri et al. 2020). A review addressing the detection of pesticides in worldwide surface waters highlighted the frequent occurrence of active ingredients of 15 insecticides, 14 herbicides, and 10 fungicides. Among these, the most frequently identified were the herbicides atrazine and metolachlor, the insecticides dimethoate and chlorpyrifos, and the fungicides tebuconazole and carbendazim (de Souza et al. 2020). In European surface waters, a comprehensive meta-analysis showed higher median concentrations for fungicides (0.96 μg L^−1^) compared to herbicides (0.063 μg L^−1^) and insecticides (0.034 μg L^−1^) (Stehle and Schulz 2015b).

Despite the widespread presence of pesticides in aquatic systems, legislation for pesticide limits in water is still very scarce, and some countries do not establish maximum residue levels for pesticides in surface waters or groundwaters. In the European Union, the Drinking Water Directive 98/83/EC sets a maximum concentration of 0.1 mg L^−1^ for each individual pesticide and their degradation products and 0.5 mg L^−1^ for the sum of all pesticides present in a sample. Furthermore, the Water Framework Directive (Directive 2000/60/EC of the European Parliament and of the Council of 23 October 2000, establishing a framework for Community action in the field of water policy) and its follow-up (Directive 2008/105/EC of the European Parliament and of the Council of 16 December 2008 on environmental quality standards in the field of water policy) set limits for a very limited subset of individual substances (priority substances and other specific pollutants), which include pesticides. Moreover, the new European Union Directive 2020/2184 for the quality of water intended for human consumption recommends a risk-based approach for pesticide monitorization, specifically emphasizing the identification of pesticides likely to be present in a given environment.

Analyzing pesticide residues in aquatic samples faces the challenges of identifying and quantifying hundreds of substances with distinct physicochemical properties. Given that pesticide concentrations in water are commonly low, at levels of ng L^−1^ to µg L^−1^, employing ultra-sensitive analytical methods is essential. Thus, analytical methods based on advanced separation and detection techniques such as gas chromatography (GC) or liquid chromatography (LC) along with mass spectrometry (MS) are the conventional and most frequently used tools for identification and quantification of pesticides in aquatic environments (Vasiljević et al. 2012). Analysis of environmental water samples with these techniques often requires a previous concentration step to increase the amount of analyte and method sensitivity. More complex water samples (e.g., wastewaters, aquaculture waters) require an additional clean-up step to eliminate interferences. Therefore, sample preparation is a critical limiting step of the entire analytical process (Campanale et al. 2021). Various sample preparation methods have been used for analysis of organic pollutants in water samples, but require the use of toxic organic compounds, and thus environmentally safe solventless methods have been pursued (Vasiljević et al. 2012; Samsidar et al. 2018; Campanale et al. 2021).

The analytical method selected is based on characteristics of the compounds analyzed. GS-MS has been used as the main analytical technique for multi-residue analysis of non-polar and volatile pesticides like organochlorines. In contrast, products with more polar structures and low volatility, such as herbicides, fungicides, and some insecticides, have been analyzed using liquid chromatography coupled to tandem mass spectrometry (LC–MS-MS) as the preferred analytical method (Bhadekar et al. 2011; Vasiljević et al. 2012; Campanale et al. 2021). Although these methods are reliable and extremely sensitive, offering low detection limits (ng L^−1^), they present several drawbacks. One of the biggest gaps in the current analytical methods for pesticide monitoring is the lack of real-time and continuous surveillance systems. Current methodologies often rely on laboratory analysis that is expensive and time-consuming, delaying real-time assessments and timely interventions. Furthermore, most conventional methods require extensive sample preparation using solvents, making them expensive and misaligned with green chemistry principles. The reliance on highly trained technicians and complex procedures further limits implementation in low-tech local laboratories, hindering broader adoption and accessibility. Furthermore, monitoring programs based on current analytical methods are geographically limited, focusing on specific regions or high-risk areas, leaving vast agricultural and natural landscapes unmonitored and failing to capture seasonal fluctuations and long-term trends in pesticide pollution. A further limitation is the tendency of many pesticide monitoring initiatives to focus on a limited set of well-known active ingredients, often neglecting newly developed pesticides and their transformation products, thus preventing a comprehensive assessment of the full spectrum of pesticide pollution (Bhadekar et al. 2011; Daverey et al. 2019; Rani et al. 2021). To address these challenges, newer and more expedite methodologies, capable of detecting an increasing number of relevant analytes in a cost-effective and efficient manner, are being developed (Rodriguez-Mozaz et al. 2006). Ideally, fast, disposable, and cost-effective detection systems are vital to meet the high-throughput demands of extensive monitoring programs. In this context, biosensors have demonstrated a great potential as a complementary tool for detection and quantification of environmental contaminants. Their integration into environmental monitoring programs could enhance surveillance capabilities, improve response times to contamination events, and ultimately contribute to better protection of ecosystems and public health.

## Biosensors as tools to screen pesticides in water

**Fig. 1:**
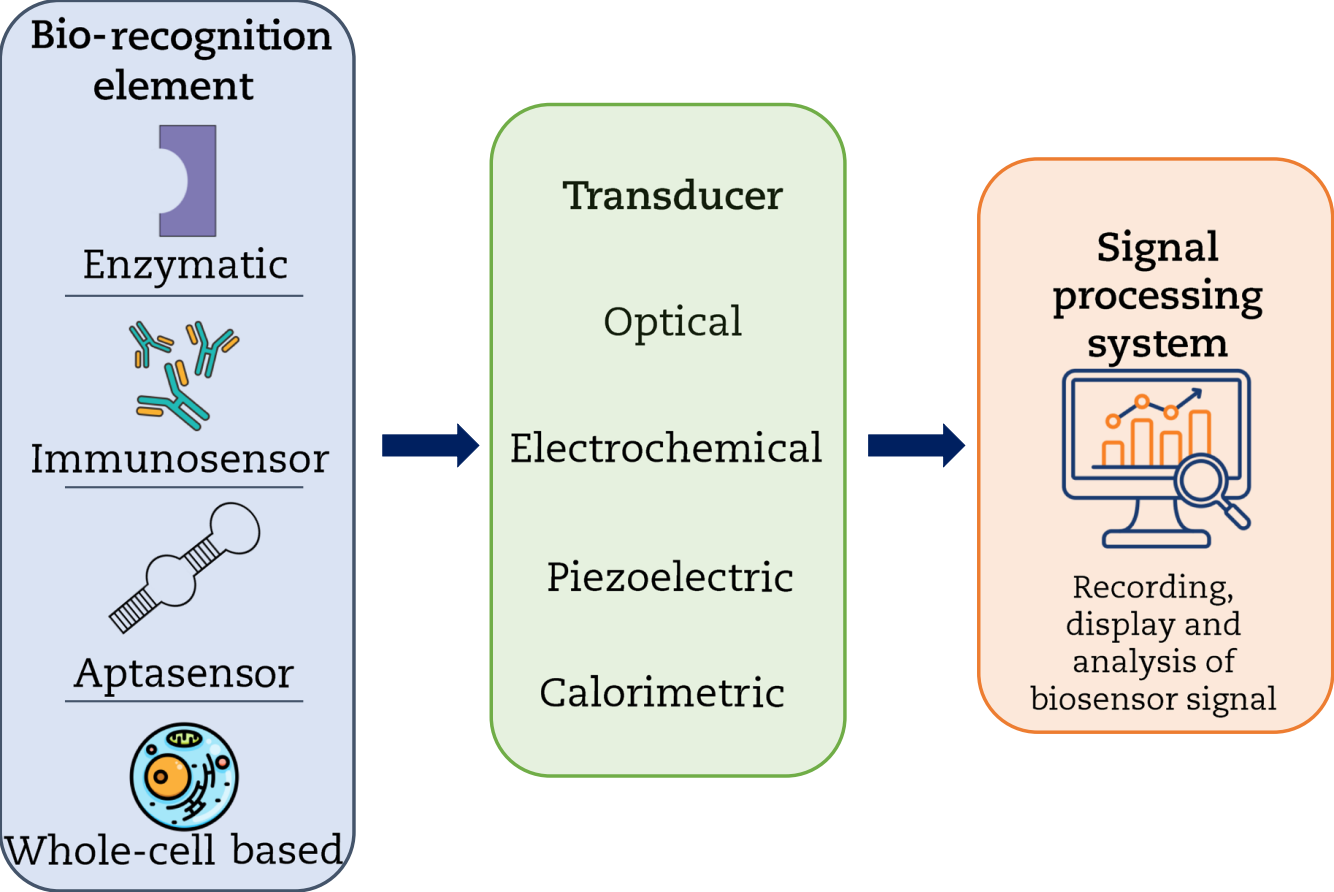
General structure of a biosensor. Target analyte is recognized by the biological recognition element, leading to an alteration that is detected and converted into a measurable signal by the transducer

According to the International Union of Pure and Applied Chemistry (IUPAC), a biosensor is a self-contained integrated device capable of measuring chemical and biological reactions and providing semi-quantitative or quantitative information.^1^ Biosensors operate on the principle of specific biological-recognition and contain three main features: a bio-recognizing element, a transducer, and a signal processing system (Fig. 1). The bio-recognition element interacts with the sample target analyte, generating a biochemical response that is subsequently converted into a quantifiable signal by the transducer element; then, the processing system translates this signal into a readable format. The generated signal is therefore related to the concentration of the analyte in the reaction (Rodriguez-Mozaz et al. 2006; Upadhyay and Verma 2015). Generally, biosensors are classified according to the transduction method used for detection or the biological receptor used as a recognition element. According to the former, biosensors can be classified as optical, electrochemical, piezoelectric, or calorimetric. Most biosensors reported in the literature are electrochemical or optical. According to the bio-recognition element, biosensors can be classified as enzymatic, immunosensors, aptasensors, or whole cell–based (Sassolas et al. 2012; Justino et al. 2015; Verma and Bhardwaj 2015). In recent years, the incorporation of nanotechnology and nanomaterials into biosensors has revolutionized and accelerated their development. The use of nanotechnology and nanomaterials enhances selectivity, sensitivity, and performance, rendering analysis more efficient, easy, fast, and economical, with improved accuracy and robustness (Berkal and Nardin 2023). Diverse nanomaterials such as metal nanoparticles (e.g., silver and gold nanoparticles), graphene and graphene-based nanocomposites, carbon nanotubes, quantum dots, and magnetic nanoparticles, among others, are therefore used increasingly often in biosensors as sensing elements (Hassani et al. 2017; Mirres et al. 2022).

Biosensors are simple in operation and less labor-intensive than chemical methods, enabling minimal sample preparation. Moreover, they offer the possibility of portability, miniaturization, and on-site detection, and are faster and more cost-effective than conventional methods (Verma and Bhardwaj 2015; Salouti and Derakhshan 2020). One of the main advantages of biosensors is their capability to provide a comprehensive analysis of the toxicity of environmental samples. Several compounds can lead to biochemical and physiological alterations in biosensors. Therefore, by analyzing metabolic and enzymatic alterations, respiratory rates, and DNA damage, among other cellular functions, researchers can gain valuable insights into how environmental samples affect biological systems. While this may present a drawback in terms of specificity, it ultimately enables a more accurate evaluation of environmental risks and facilitates the extrapolation of toxicity effects to more complex organisms, contributing to improved ecological and human health assessments (Gui et al. 2017). Biosensors have been employed in different fields such as food quality monitoring, pathogen and drug discovery, disease detection, evaluation of toxicity, and environmental monitoring (reviewed in Lozano et al. (2019); Haleem et al. (2021); Gavrilaș et al. (2022); Huang et al. (2023)). The latter encompass biosensors that detect a variety of pollutants such as pesticides, phenols, metals, and endocrine-disrupting chemical compounds. In this review, we provide an overview of biosensors that have been specifically developed for environmental water monitoring of different classes of pesticides (Table 1).

**Table 1:**
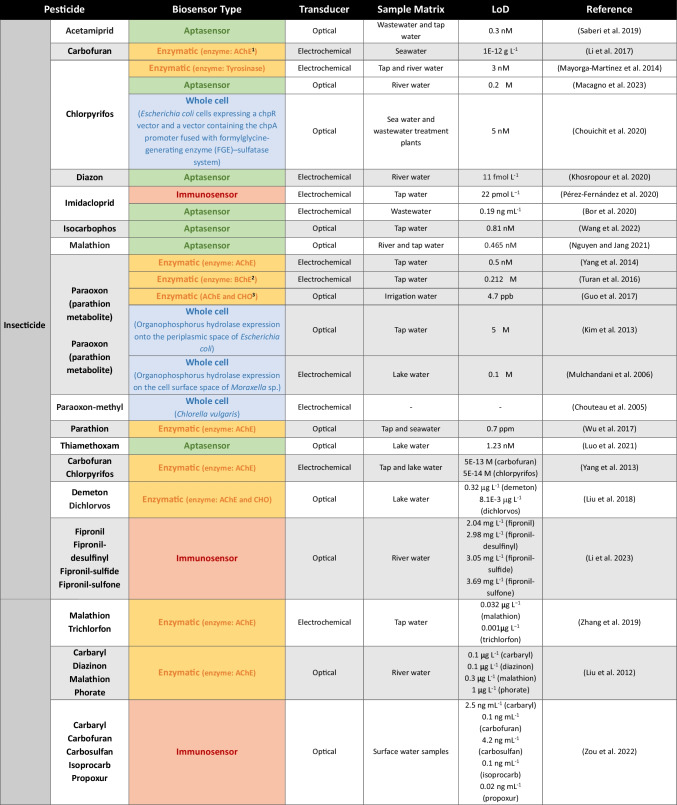

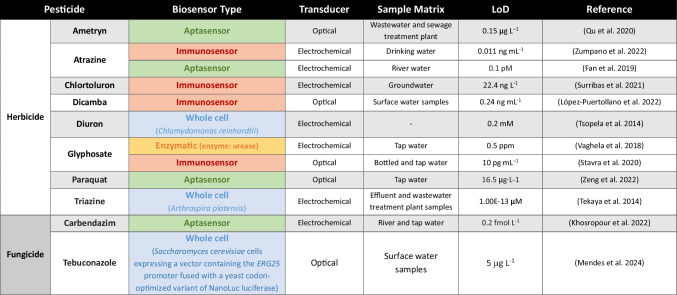
Examples of biosensors for environmental water monitoring of different classes of pesticides. For each example, the pesticide class (insecticide, herbicide, or fungicide), pesticide name, the biosensor type (enzymatic, immunosensor, aptasensor, or whole cell), transducer, sample matrix used to test biosensor applicability and limit of detection (LoD) are indicated

^1^*AChE* acetylcholinesterase,^2^*BChE* butyrylcholinesterase, ^3^*CHO* choline oxidase

### Enzymatic biosensors

**Fig. 2:**
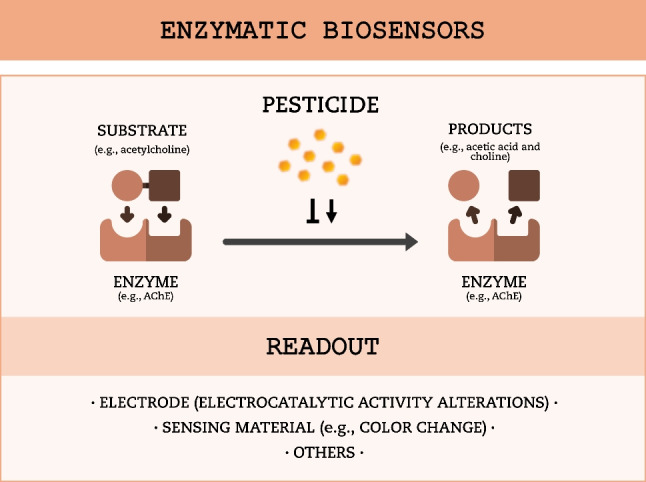
Schematic representation of enzymatic biosensors. Enzymatic activity is stimulated or (more common) inhibited by the pesticide, leading to increase or decrease of the product, which can be monitored through spectrophotometry, electrochemistry, or other detection methods

Enzymatic biosensors or enzyme-based biosensors were the first class of biosensors to be developed. In 1962, Clark and Lyons developed the first “true” biosensor, for glucose detection, by incorporating the enzyme glucose oxidase into a Clark oxygen electrode (Clark and Lyons 1962). Since then, enzymatic biosensors have been the most used and well-studied type of biosensors (Kucherenko et al. 2019). In enzymatic biosensors, an enzymatic reaction is affected by the target analyte, and alterations in levels of a product or substrate are then detected (Chadha et al. 2022) (Fig. 2). Enzymatic biosensors are rapid, user-friendly, and easily automated, facilitating scalability. Moreover, they are very sensitive, allowing for detection of analytes even at low concentrations. This can be explained by the high catalytic activity and wide availability of enzymes for a variety of functions and substrates (Rebollar-Pérez et al. 2015; Alvarado-Ramírez et al. 2021; Hara and Singh 2021). However, enzyme production is costly and enzymes have limited stability, as they are susceptible to degradation or denaturation, which affects biosensor performance and longevity. Additionally, enzymatic activity can be influenced by the presence of various compounds, pH, temperature, and other environmental conditions, affecting biosensor selectivity, accuracy, and reliability (Luque de Castro and Herrera 2003; Alvarado-Ramírez et al. 2021).

In pesticide biosensing, the cholinesterase group is the most widely used enzyme group, particularly acetylcholinesterase (AChE). This enzyme is sensitive to a broad spectrum of chemical compounds, including organophosphate and carbamate pesticides. Hence, AChE is a pivotal component in biosensing assays targeting these compounds (Sassolas et al. 2012; Štěpánková and Vorčáková 2016). For instance, Yang et al. (2014) developed an enzymatic biosensor for the amperometric detection of paraoxon, a metabolite of the organophosphorus insecticide parathion. AChE was encapsulated into a silica matrix immobilized onto a glassy carbon electrode modified with gold nanoparticles, polypyrrole, and reduced graphene oxide sheets. The developed biosensor was tested in solutions containing the substrate (acetylthiocholine), in the presence or absence of paraoxon. In samples without paraoxon, acetylthiocholine is hydrolyzed by AChE into thiocholine, resulting in a higher electrocatalytic activity due to its oxidation. In contrast, the enzymatic reaction is inhibited in the presence of paraoxon, leading to a reduced electrocatalytic activity. Biosensor activity was further tested in tap water samples spiked with paraoxon, yielding a detection limit of 0.5 nM. Along with AChE, butyrylcholinesterase (Turan et al. 2016), tyrosinase (Mayorga-Martinez et al. 2014), and urease (Vaghela et al. 2018) have also been used in enzymatic biosensors to monitor pesticides in environmental water samples. Generally, only one enzyme is used, but the combined use of several enzymes can yield better results in some cases, broadening the spectrum of detected analytes. A recent work developed a biosensor composed of two enzymes, AChE and choline oxidase (CHO), and gold nanorods. Briefly, the substrate acetylcholine is degraded by AChE to produce choline. Subsequently, choline is oxidized by CHO into betaine and into the oxidizing agent H₂O₂, which triggers the chemical etching of gold nanorods (AuNRs) and induces a decrease in their aspect ratio. The etching of the AuNRs leads to distinct colors that are recorded with a UV spectrophotometer. Since the activity of AChE is inhibited by organophosphorus pesticides, leading to a reduction in H₂O₂ production and the prevention of AuNRs etching, they can be detected. Biosensor applicability was tested in lake water samples spiked with dichlorvos and demeton, and detection limits of 8.1 × 10^−3^ mg L^−1^ and 0.32 mg L^−1^, respectively, were observed (Liu et al. 2018). More examples of enzymatic biosensors, specific for environmental water monitoring of pesticides, are summarized in Table 1, depicted in orange.

### Immunosensors

**Fig. 3:**
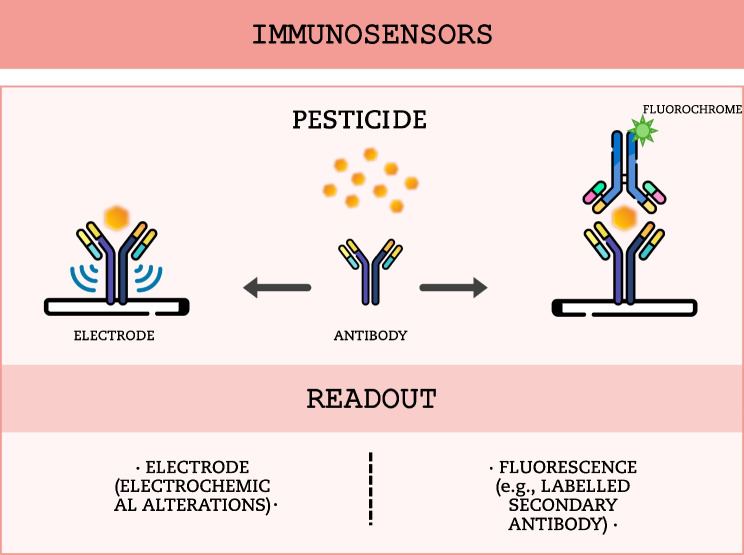
Schematic representation of immunosensors. The pesticide function as the antigen, binding to the antibody and thus triggering a response signal via an antibody-bound transducer (e.g., electrode, left: Label-free immunosensor) or signal-producing labels (Labeled immunosensor, right)

Immunosensors, also called antibody-based biosensors, depend on the reaction between an antigen and an antibody; thus, the bio-recognition element is the antigen–antibody complex. An antigen is a molecule that elicits an immune response and can combine with the specific antibody. The formation of this complex generates a read-out signal further detected by a transducer (Jain et al. 2019; Gavrilaș et al. 2022). The first step in developing an immunosensor is the production and isolation of antibodies to the desired analyte. Then, they need to be integrated into an assay system that generates a visible reaction (Hartzler 1990; Reynoso et al. 2019; Fang et al. 2020) (Fig. 3).

Immunosensors are generally classified into label-free and labeled sensors. Label-free immunosensors measure the physical changes resulting from the antigen–antibody complex formation. The binding event can lead to alterations in mass, refractive index, conductivity, or other characteristics, which are then detected and transduced into measurable signals by the sensor system (Fig. 3 left). In contrast, labeled immunosensors use signal-producing labels (such as enzymes, fluorescent dyes or electroactive compounds) attached to the target analyte or the bioreceptor so that the activity of the marker is linked to the amount of analyte, allowing for a more sensitive and versatile detection (Fang et al. 2020; Gavrilaș et al. 2022) (Fig. 3 right).

Immunosensors provide an easy, rapid, and high-throughput method for pesticide detection with greater accuracy, specificity, and sensitivity than traditional methods. However, the production of antibodies is laborious, highly costly, and challenging, and alterations in temperature or pH can affect immunosensor performance (Jain et al. 2019). Moreover, antibody cross-reactivity with similar analytes is commonly observed, potentially leading to false positive results. The need to develop specialized reagents for each compound is also a weakness of immunosensors. Therefore, development of broad-specificity antibodies can be the solution. In this case, based on a similar structure of a group of molecules, an antibody with broad cross-reactivity and capable of detecting multiple analytes is produced. Antibodies with this broad detection spectrum are an attractive approach for multi-residue monitoring (Reynoso et al. 2019). For instance, Li et al. (2023) developed a broad-specificity antibody for detection of the pesticide fipronil and three major metabolites (fipronil-sulfone, fipronil-sulfide, and fipronil-desulfinyl). Then, using an antibody-based immunoassay (ELISA), the authors were able to detect fipronil and its metabolites in river water with a detection limit varying from 2.04 to 3.69 mg L^−1^, depending on the metabolite. More examples of immunosensors for environmental water monitorization of pesticides are presented in Table 1, depicted in red.

### Aptasensors

Aptasensors, also known as aptamer-based biosensors, use aptamers as bio-recognition elements. An aptamer is an artificial and short (10–100 nucleotides in length) single-stranded DNA or RNA sequence that displays a specific tertiary structure through folding. They are obtained by SELEX (Systematic Evolution of Ligands by Exponential Enrichment) in vitro screening, and are therefore often named “chemical antibodies” (Toh et al. 2015). The SELEX methodology involves three main steps: (i) incubation of the target analyte with a library; (ii) selection of aptamer–target complexes from unbound oligonucleotides; and (iii) amplification of the bound sequences. Briefly, a random library of single-stranded nucleic acids (DNA or RNA) is incubated with the target analytes. After incubation, unbound variants are eliminated, and the target-bound oligonucleotides are amplified through PCR and used in the following rounds of SELEX. After several rounds (8–15), sequences with a higher affinity for the target analyte become dominant in the library. Finally, the sequences are analyzed, and the most prevalent sequence is identified as the potential aptamer (Ellington and Szostak 1990; Tuerk and Gold 1990; Wilson and Szostak 1999).

**Fig. 4:**
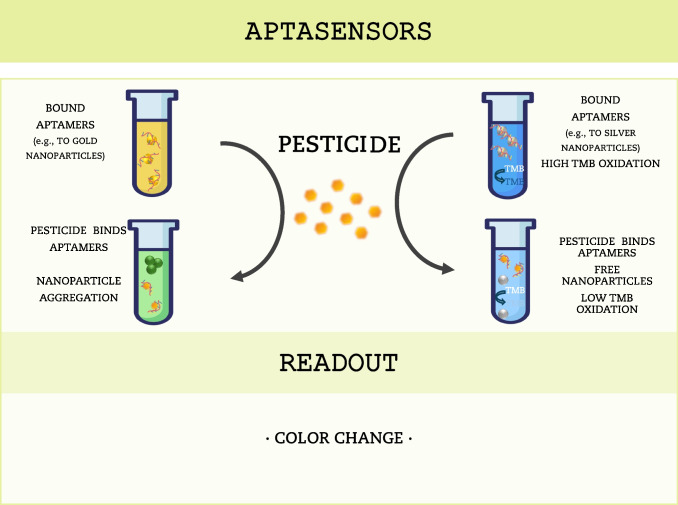
Schematic representation of commonly used color-based aptasensors. Aptasensors based on color shift due to gold nanoparticle aggregation (left) or chemical reaction (right)

Aptamers interact with their target molecules similarly to how an antigen interacts with an antibody. However, aptasensors have several advantages over other biosensors that use natural receptors such as antibodies or enzymes. Aptamers have a unique tertiary structure such as hairpins, internal loops, pseudoknots, T-shaped structures, and G-quadruplexes, allowing them to bind to any given target, ranging from small molecules to large proteins and even cells, thus allowing the development of a wide range of aptamer-based biosensors. Unlike the preparation of antibodies, which require biological systems to be generated, aptamers can be easily produced via chemical synthesis, which offers a higher specificity and affinity with the target and eliminates the use of animals, decreases batch-to-batch variations, and reduces production costs and time. Furthermore, aptamers are more structurally and functionally stable over a wide range of pH and temperatures (Song et al. 2008; Toh et al. 2015; Xie et al. 2022). Similar to immunosensors, aptasensors can also be functionalized with enzymes or fluorescent probes, and are thus further categorized as label-free or labeled aptasensors (Liu et al. 2019) (Fig. 4).

In the past few years, considerable time and effort has been dedicated to aptamer screening and aptasensor development. Thus, research in this field has significantly expanded, culminating in the development of aptasensors for pesticide monitoring. Several aptamers have already been developed to selectively recognize various types of pesticides, such as organochlorines, organophosphates, neonicotinoids, and others (reviewed in Qin et al. (2023)). For example, Zeng et al. (2022) developed an aptamer-based biosensor to detect the herbicide paraquat, based on the oxidation of 3,3′,5,5′-tetramethylbenzidine (TMB) by Ag^+^. In the absence of paraquat, the paraquat-aptamer (PQ-15) binds to Ag^+^; as the electron transfer from the aptamer-Ag^+^ complex to dissolved oxygen increases, the release of superoxide anion radicals (O_2_^−^) is enhanced and facilitates the oxidation of TMB, resulting in a dark blue solution. On the other hand, in the presence of paraquat, the PQ-15 aptamer preferentially binds to the pesticide to form a more stable complex. This decreases the catalytic activity of Ag^+^, leading to less TMB oxidase and a light blue or colorless solution. The absorbance of solutions is recorded with a UV/VIS spectrophotometer (Fig. 4, right). The authors tested the aptasensor selectivity for other pesticides, and found it was specific for paraquat, with a detection limit of 16.5 μg L^−1^. A color shift based on gold nanoparticle aggregation is also widely used in aptasensors (Bai et al. 2015; Qu et al. 2020; Macagno et al. 2023). Briefly, in the absence of the target molecules, aggregation is prevented because gold nanoparticles bind to aptamers in the presence of a destabilizing agent (e.g., NaCl). In contrast, in the presence of the analyte, aptamers dissociate from gold nanoparticles to bind to their specific target, leading to gold nanoparticle aggregation and color change, even in the presence of a destabilizing agent. The absorbance of solutions is measured with a UV/VIS spectrophotometer (Macagno et al. 2023) (Fig. 4, left). Additional examples of aptasensors for environmental monitoring of pesticides in aqueous samples are displayed in Table 1, highlighted in green.

### Whole cell–based biosensors

**Fig. 5:**
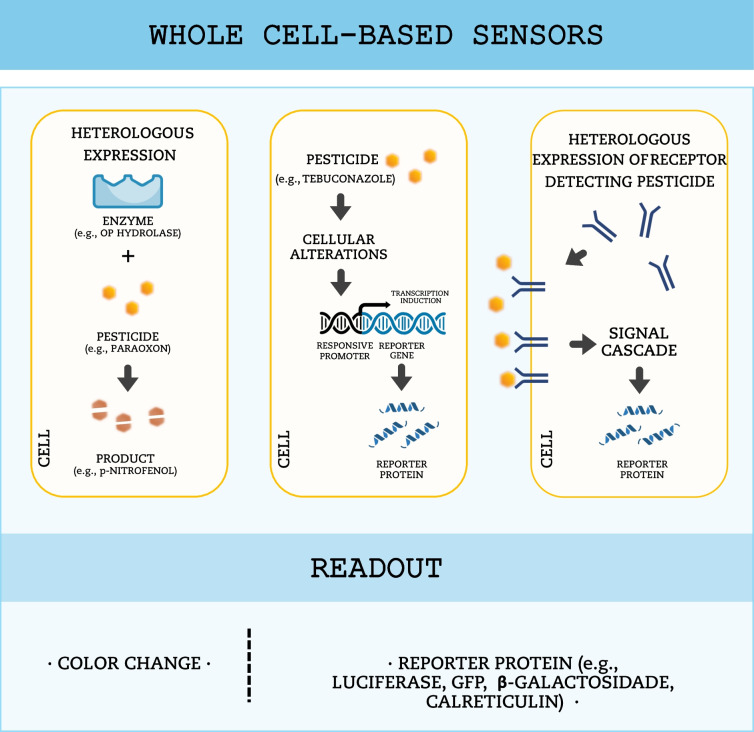
Schematic representation of whole cell–based biosensors. Whole cells work as biorecognition elements and may be engineered to enhance the readout (e.g., reporter genes inserted in custom-made “operons”). Pesticides bind to membrane receptors or are internalized and interact with enzymes or other cellular constituents, triggering signaling cascades or causing cellular alterations that can be detected though a readout signal

Instead of isolated molecules, such as enzymes, antibodies, or aptamers, living cells can also be used as biorecognition elements, in so called whole cell–based biosensors. Different types of cells have been used in biosensor development, stemming from bacteria and fungi to higher eukaryotes like fish, rats, and human cells. Algal and microbial cells (namely bacteria, filamentous fungi and yeast) are predominantly used in biosensors for environmental monitoring and to assess toxicity of environmental contaminants. In contrast, higher eukaryotic cell lines play significant roles in exploring fundamental cellular processes and understanding disease development (Gupta et al. 2019). Whole cell–based biosensors can operate in two ways: by employing the inherent ability of a particular organism to respond to specific substances or by incorporating engineered genetic constructs into the organism to detect bioavailable analytes (Gupta et al. 2019; Moraskie et al. 2021) (Fig. 5). Biosensors using the natural sensing abilities of cells offer advantages in detecting a variety of compounds. However, as they are more vulnerable to interference from other species, biosensors based on engineered cells are often used (Moraskie et al. 2021). Of note, is important to differentiate between biosensors and bioassays based on whole cells. A biosensor, according to the IUPAC definition mentioned above, implies a device that converts a biological response into a quantifiable signal. In contrast, a bioassay implies an observable response to the presence of a target compound, but without direct transduction into a readout, instead using general physiological changes as endpoints, such as growth inhibition or morphological alterations.

Whole cell systems provide a reliable alternative to molecular biosensors, as cells are relatively easy to develop and can metabolize a wide range of chemical compounds, since the necessary cofactors are already present intracellularly. Thus, there is no need to isolate subcellular components like enzymes or proteins. Moreover, microbial cells for biosensors can be massively produced through simple culturing steps, and have a higher stability over a wide range of environmental changes, including alterations in temperature and pH, and can be modified through simple genetic engineering methods (Park et al. 2013; Gui et al. 2017; Plekhanova and Reshetilov 2019). Additionally, this type of biosensors can not only detect specific analytes but also offer additional information that molecule-based biosensors cannot provide, such as information related to the pharmacology, cell physiology alterations, and toxicology of the sample (Gui et al. 2017). For instance, yeast-based biosensors and bioassays have been successfully applied for toxicity and genotoxicity assessments of water samples, using yeast strains with reporter constructs responsive to genotoxic stress (Lichtenberg-Fraté et al. 2003; Knight et al. 2004; Eki 2018). Thus, while not pesticide-specific, they complement conventional analytical methods by providing crucial information about the broader toxicological impact of pesticide exposure.

Despite multiple advantages, several drawbacks can limit the use of whole cell–based biosensors, such as the need to preserve cell activity and viability under adverse environmental conditions. Moreover, when using genetically engineered cells, prolonged response times are often required for expression of the reporter gene, representing a significant limitation for real-time detection (Bilal and Iqbal 2019; Gupta et al. 2019), but still providing a good alternative to natural sensing abilities (Moraskie et al. 2021). For example, Kim et al. (2013) developed engineered cells for detection of paraoxon. They used recombinant *E. coli* cells expressing the organophosphorus hydrolase enzyme at the periplasmic space. In the presence of paraoxon, the enzyme catalyzes its hydrolysis into *p*-nitrophenol and diethylphosphoric acid, and then absorbance of *p*-nitrophenol is measured, which is directly correlated with paraoxon concentration.

Reporter gene expression under the control of a specific promoter is another powerful readout used in engineered microbial biosensors. Upon exposure to the target, the promoter is activated, inducing or increasing the expression of the reporter gene. Then, this biological response is converted into a detectable signal via a transducer (Justino et al. 2015; Gui et al. 2017; Saini et al. 2019). The typical reporter genes used in microbial biosensors are firefly luciferase, calreticulin, β-galactosidase, green fluorescent protein, and bacterial luciferase (Gu et al. 2004; Aynalem and Muleta 2021). However, other reporter genes can also be used. For instance, Chouichit et al. (2020) developed a whole cell reporter system for detection of chlorpyrifos using bacteria. First, they identified the *Sinorhizobium meliloti* chlorpyrifos-responsive promoter (chpA) as highly induced by chlorpyrifos and dependent on the chlorpyrifos-responsive transcription regulator (ChpR) (Whangsuk et al. 2010). Then, they engineered *E. coli* cells with a chpR expression vector and a plasmid encoding the chpA fused with formylglycine-generating enzyme (FGE)–sulfatase system. Therefore, when cells are exposed to chlorpyrifos, ChpR activates the expression of the FGE–sulfatase system via the chpA promoter. Then, FGE modifies a serine residue of the sulfatase, resulting in the formation of activated sulfatase (formylglycine (fGly)), which cleaves 4-methyllumbeliferone sulfate (4-MUS) to produce the fluorescent product 4-methyllumbeliferone (4-MU) (Whangsuk et al. 2016). The authors were able to detect chlorpyrifos in spiked sea water and wastewater treatment plant samples, with a detection limit of 5 nM, with the same system but using a plasmid that overexpressed ChpR (Chouichit et al. 2020).

Along with the microbial biosensors that use bacteria as biorecognition elements, yeast-based systems, especially those using *S. cerevisiae*, are a promising alternative (Adeniran et al. 2015; Chamas et al. 2017). Indeed, biosensors based on the yeast *S. cerevisiae* model have already been used to detect endocrine disruptive substances (Sanseverino et al. 2005; Cevenini et al. 2018; Lobsiger et al. 2019). Nonetheless, despite its potential and the invaluable add-on to the available analytical toolbox, yeast-based biosensing is still underexplored. Recently, an engineered yeast-based biosensor was developed for environmental monitoring of tebuconazole, a common agrochemical fungicide (Mendes et al. 2024). The biosensor is based on the expression of a reporter gene downstream of the *ERG25* promoter, which is induced by ergosterol depletion caused by tebuconazole. In this work, a yeast codon-optimized variant of nanoluciferase was used as a reporter gene so that, upon addition of the luciferase substrate, tebuconazole can be detected by measuring bioluminescence. This biosensor displayed a detection limit of 5 μg L^−1^ and was validated in spiked natural water samples. More examples of whole cell–based biosensors, specific for environmental monitoring of pesticides in aqueous samples, are summarized in Table 1, highlighted in blue.

## Conclusions and perspectives

Biosensors represent a promising technology for environmental monitoring of pesticides in aquatic systems, offering several advantages. These include simple operation and limited sample preparation, making them less labor-intensive than chemical methods. Moreover, biosensors are highly sensitive and specific, and often portable, enabling on-site detection and providing real-time results. Finally, biosensors are cost-effective and environmentally friendly compared to traditional analytical methods.

Despite their promising capabilities, the widespread adoption of biosensors encounters several challenges that have hindered their commercial application. A significant number rely on biological components such as enzymes and antibodies, which are inherently prone to degradation or loss of activity over time. This short shelf life limits their practical application for environmental monitoring. Additionally, environmental factors such as pH fluctuations, temperature variations, salinity changes, and the presence of multiple contaminants can lead to inconsistent readings, ultimately reducing biosensor accuracy and reliability (Luque de Castro and Herrera 2003; Jain et al. 2019; Alvarado-Ramírez et al. 2021). Beyond stability concerns, biosensors often lack the capability for simultaneous detection of multiple contaminants. Moreover, most biosensors were developed for detection of insecticides, with relatively few developed for herbicides or fungicides, despite the fact that global sales of herbicides and fungicides exceed those of insecticides (Pimentão et al. 2024). Expanding the range of compounds detected by biosensors could therefore lead to more comprehensive monitoring of environmental contaminants.

Perhaps more importantly, the use of biosensors within the regulatory frameworks remains limited. Indeed, very few biosensors have received official recognition for regulatory use. A notable exception is the use of the yeast estrogen screen (YES) and yeast androgen screen (YAS) bioassays as validated methods for assessing the estrogenic potential of water and wastewater using genetically modified *S. cerevisiae*, which has been approved by the International Organization for Standardization (ISO). For biosensors to gain regulatory acceptance, new methods must undergo rigorous validation by regulatory agencies to ensure reliability, reproducibility, and robustness. Conventional techniques have well-established validation protocols, well-defined calibration standards, and reference materials, making them the preferred choice for regulatory applications. In contrast, biosensors lack universally accepted protocols for performance evaluation. The absence of standardized regulatory pathways and universally accepted performance benchmarks remains a significant challenge to their official approval (Lindquist 2020; Nath 2024). Altogether, these factors contribute to the limited commercial application of biosensors for environmental water monitoring of pesticides. To facilitate the widespread adoption of biosensors in environmental water monitoring, it is essential to develop strategies that enhance their long-term stability while ensuring optimal accuracy and reliability across diverse environmental conditions. Further standardization efforts should prioritize closer collaboration between biosensor developers and regulatory agencies to establish universal calibration protocols and clearly defined performance criteria (Nath 2024).

In conclusion, considering the advantages and disadvantages of biosensors, they are best positioned as complementary tools to conventional methods in a tiered assessment approach, serving as a time- and cost-effective screening step before more detailed analytical methods. This strategy would ultimately facilitate semi-continuous environmental analyses, enhancing monitoring efficiency. Therefore, further efforts should be directed toward the adoption and integration of biosensors into environmental monitoring programs. Addressing these challenges through continued research and development efforts will be crucial to maximize the potential of biosensors for pesticide monitoring and ensuring improved environmental safety.

## Footnotes

1 https://goldbook.iupac.org/terms/view/B00663 (assessed on 15 February 2025).
